# Enhanced SARS-CoV-2 Antibody Response After a Third Heterologous Vector Vaccine Ad26COVS1 Dose in mRNA Vaccine-Primed Kidney Transplant Recipients

**DOI:** 10.3389/ti.2022.10357

**Published:** 2022-03-22

**Authors:** Judith Schimpf, Tamara Davidovic, Armin Abbassi-Nik, Hannelore Sprenger-Mähr, Karl Lhotta, Emanuel Zitt

**Affiliations:** ^1^ Department of Internal Medicine 3 (Nephrology and Dialysis), Academic Teaching Hospital LKH Feldkirch, Feldkirch, Austria; ^2^ Vorarlberg Institute for Vascular Investigation and Treatment (VIVIT), Feldkirch, Austria; ^3^ Agency for Preventive and Social Medicine (aks), Bregenz, Austria

**Keywords:** Covid-19, Sars-CoV-2, kidney transplantation, Ad26COVS1, heterologous vaccination

Dear Editors,

We and others have shown that kidney transplant recipients (KTR) exhibit a reduced immune response with a seroconversion (SC) rate <50% after a regular 2-dose mRNA SARS-CoV-2 vaccination regimen (1, 2). Very limited data on a heterologous 3-dose vaccination with the vector vaccine Ad26COVS1 are available in this patient group. In the only published trial, a 3-dose homologous vaccination protocol was compared with a heterologous one in KTR without SC after a 2-dose mRNA vaccination (3). The third dose increased the antibody response and was well tolerated. However, still less than 50% of the initial non-responders developed SC 4 weeks after either a third mRNA vaccine (35%) or the vector vaccine Ad26COVS1 (42%) (3).

Herein, we provide additional data on the humoral response in 142 Austrian KTR (mean age 60.6 years, 59.9% male, median transplantation vintage 109 months) after double mRNA and triple heterologous vaccination (Figure 1). Patients provided written informed consent, and the study was conducted in compliance with the Helsinki Declaration of 1975, as revised in 2013. Out of 122 patients with follow-up, 76 patients being vaccinated with two doses of a mRNA vaccine (75% mRNA-1273, 25% BNT162b2) received a third dose of Ad26COVS1, administered on average 109 days (range 109.0–145.0 days) after the second dose. SC was determined on average 47 days (range 35.5–61.0 days) after the third vaccination by quantifying anti-SARS-CoV-2 spike IgG antibodies (LIAISON^®^ SARS-CoV-2-TrimericS IgG chemiluminescent immunoassay, Diasorin S.p.A., Saluggia, Italy; cut-off value for seroconversion: ≥33.8 BAU/mL). After double mRNA vaccination the SC rate was 48%. Following heterologous triple vaccination an additional 54% of the initial non-responders achieved SC. Altogether, 97 out of 122 KTR (80%) achieved SC after either double mRNA vaccination or the heterologous triple vaccination. Forty-eight of the 142 KTR showed high-level SC after double mRNA vaccination. Twenty patients developed low antibody concentrations (arbitrary threshold <350 BAU/mL). After a third heterologous dose all these 20 patients significantly boosted their humoral response (1391.9 (SD 687.2) *vs*. 144.8 (SD 94.6) BAU/mL, *p* < 0.001). Non-responders after heterologous triple vaccination were significantly older (65.5 *vs*. 59.4 years; *p* = 0.033), were more often treated with prednisolone or belatacept (88% *vs*. 46.4%, 28% *vs*. 2.1%; *p* < 0.001 for both) and had a shorter median transplantation vintage (66.0 *vs*. 141.7 months; *p* < 0.001). They showed a trend of lower mean eGFR (48.1 *vs*. 55.5 ml/min/1.73 m^2^; *p* = 0.058) and being treated more often with mycophenolic acid (84% *vs*. 64%; *p* = 0.090). Higher mycophenolic acid doses did not correlate with inferior antibody response (*p* = 0.299). As a limitation, our study lacks cellular immune response and neutralizing antibody data. But anti-spike IgG antibodies are highly correlated with neutralizing antibodies, and a level >264 BAU/mL (95% CI: 108, 806) has been found to be associated with 80% vaccine efficacy against primary symptomatic Covid-19, although limited to the B.1.177 and B.1.1.7 SARS-CoV-2 variant (4). Fifty-three percent (40/76) of our patients with a third heterologous dose achieved this threshold, 62% of those with SC after initial non-response (18/29). Whether this threshold indicates the same vaccine efficacy against the now dominant SARS-CoV-2 Omicron variant is unknown. The longer transplantation vintage (9.0 *vs*. 4.6 years) and extended interval between second and third dose (109 *vs*. 80 days) in our cohort compared to the study by Reindl-Schwaighofer et al. (3) might be responsible for the higher seroconversion rate in our heterologous prime-boost vaccinees, as both factors significantly influence the vaccination response (1, 5). Nevertheless, due to our study design we cannot recommend one vaccine platform as superior over the other for booster vaccination in KTR, a clinically relevant question addressed by others (3). It remains to be proven whether a heterologous prime-boost regimen combining mRNA and vector vaccine improves the neutralizing humoral response against the now dominant SARS-CoV-2 Omicron variant in KTR as has been shown in the general population (6) or enhances the variant-specific cellular immune response (7) which might translate into better clinical outcomes.

**FIGURE 1:**
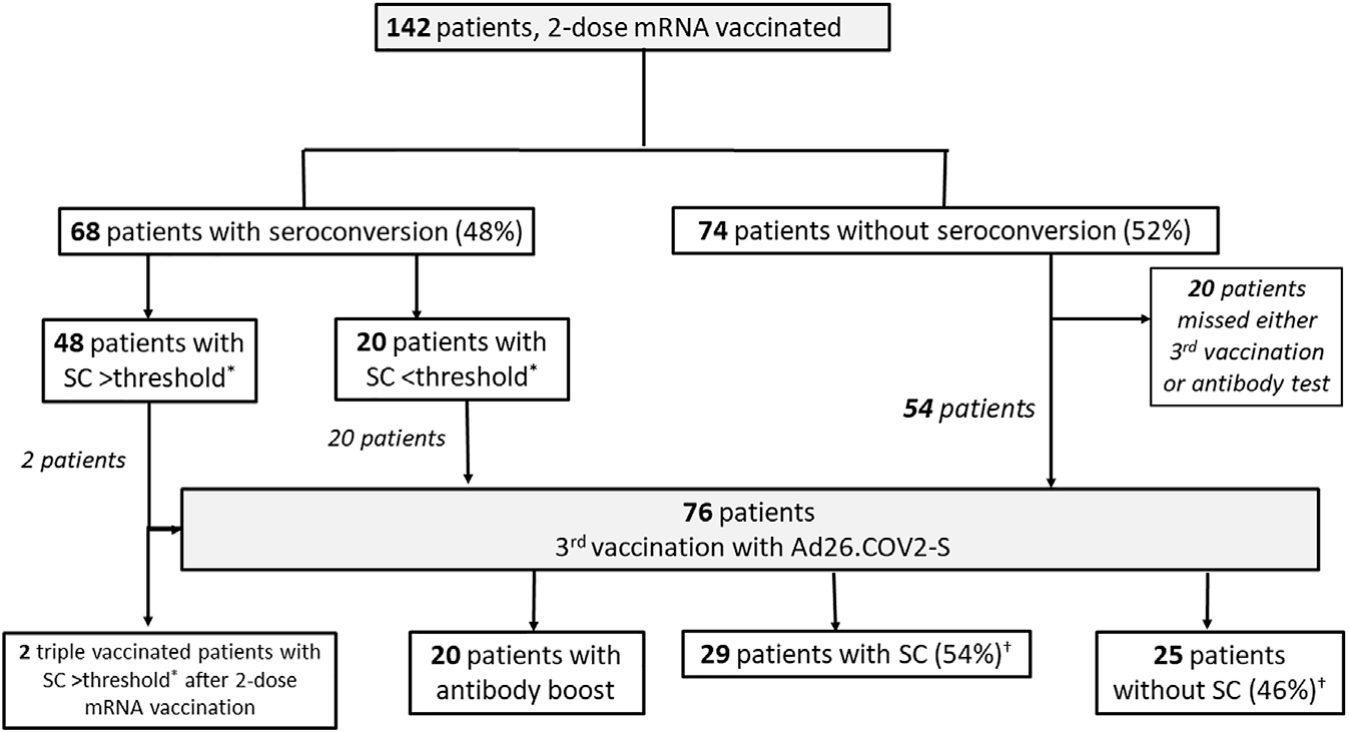
Patient Flow Chart. *Seroconversion (SC) >threshold defined by anti-SARS-CoV-2 spike protein IgG antibodies >350 BAU/mL (LIASION^©^ anti-SARS-CoV-2 TrimericS IgG chemiluminescent immunoassay, Diasorin S.p.A., Saluggia, Italy; cut off value for seroconversion: ≥33.8 BAU/mL). ^†^% of patients (*n* = 54) without initial seroconversion after two mRNA doses.

## Data Availability

The original contributions presented in the study are included in the article/Supplementary Material, further inquiries can be directed to the corresponding author.
